# New Glycosides from the Fruits of *Nicandra physaloides*

**DOI:** 10.3390/molecules22050828

**Published:** 2017-05-17

**Authors:** Yan Liu, Hai-Bing Jiang, Zhen-Peng Xu, Yan-Gang Cheng, Shao-Wa Lv, Bing-You Yang, Hong-Wei Guo, Hai-Xue Kuang

**Affiliations:** Key Laboratory of Chinese Materia Medica (Ministry of Education), Heilongjiang University of Chinese Medicine, Harbin 150040, China; lifeliuyan@163.com (Y.L.); m15822237055@163.com (H.-B.J.); xu872821578@163.com (Z.-P.X.); chengyg1992@163.com (Y.-G.C.); lswa5599@hotmail.com (S.-W.L.); ybywater@163.com (B.-Y.Y.)

**Keywords:** *Nicandra physaloides*, phenyl-glycosides, fat glycosides, RAW 264.7, inflammation

## Abstract

Three new glycosides (**1**–**3**) and 15 known ones (**4**–**18**) were isolated and identified from the fruits of *Nicandra physaloides*. The structures of these compounds were established by 1D and 2D NMR spectra and HR-ESI-MS. The compounds (**4**–**18**) were the first time isolated from the *Nicandra* genus and they (except **8**, **10**, **14**) exhibited inhibitions on the NO release of LPS-induced RAW 264.7 cells with IC_50_ values from 26.9 to 47.5 μM.

## 1. Introduction

**Figure 1:**
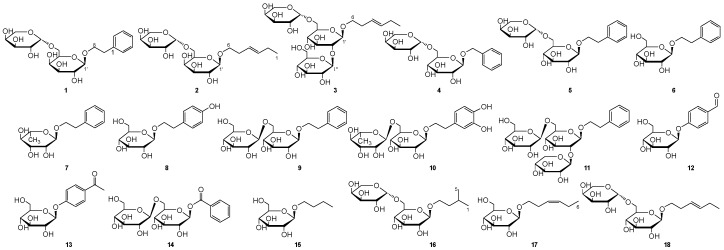
Structures of compounds **1**–**18** from *Nicandra physaloides.*

*Nicandra physaloides* (L.) Gaertn (Solanaceae) was recorded in the Dictionary of Medicinal Plants [1], as being an annual herb of monotypic genus, sweet and acerbity, bitter and natural in flavor, which possessed the various effects of sedation, eliminating phlegm, heat-clearing and detoxifying. Modern researchers have indicated that *Nicandra physaloides* was reported being taken as analgesic, vermifuge, antibacterial agent, antipyretic, diuretic and mydriatic [2,3,4] and applied to the treatments of hydrophobia, psychosis, epilepsy, rheumatoid arthritis, nasosinusitis, influenza, urinary tract infection, sore and furuncle [5,6]. Meanwhile, it was used in folk medicine for sedation, eliminating phlegm, heat-clearing and detoxifying. Equally important, the Chinese Herbal Medicine Anthology of Yun Nan recorded that the fruits of *Nicandra physaloides* (L.) Gaertn possessed the effects of dispelling wind and anti-inflammation. However, the effective material basic was unclear and indistinct [7,8]. As a part of our ongoing research on structurally diverse and anti-inflammatory ingredients from *Nicandra physaloides*, our study led to the isolation of three new compounds, Nicglycoside A–C (**1**–**3**), along with fifteen known ones including benzyl α-l-arabinopyranosyl-(1→6)-β-d-glucopyranoside (**4**) [9], 6-*O*-l-arabinopyranosyl-β-d-glucopyr-anoside (**5**) [10], 2-Phenylethyl-β-d-glucopyranoside (**6**) [11], (+)-phenylethyl-8-*O*-α-l-rhamnopyr-anoside (**7**) [12,13], Salidroside (4-hydroxy-2-Phenylethyl-*O*-β-d-glucopyranoside, **8**) [14], phenethanol-β-d-gentiobioside (**9**) [15], Forsythoside E (3,4-dihydroxyl-2-Phenylethyl-*O*-β-l-arabinopyranosyl-(1→6)-β-d-glucopyranoside, **10**) [16], and phenethylalcohol 8-*O*-β-d-glucopyranosyl-(1→2)-[*O*-α-l-arabinopyranosyl-(1→6)]-*O*-β-d-glucopyranoside (**11**) [17], Helicide (4-β-d-galactopyranosyloxy-benzaldehyde, **12**) [18], picein (1-[4-(β-d-glucopyranosyloxy-)phenyl]-ethanone, **13**) [19], benzoic acid-β-d-gentiobioside (**14**) [14], butyl-β-d-glucopyranoside (**15**) [20], Foliachinenoside I (3-methylbutyl-4-*O*-α-l-arabinopyranosyl(1→6)-β-d-glucopyranoside, **16**) [21], (*Z*)-3-Hexenyl-1-*O*-β-d-glucopyranose (**17**) [22] and (*Z*)-3-hexenyl *O*-α-arabinopyranosyl-(1→6)-*O*-β-d-glucopyranoside (**18**) [23], respectively (Figure 1, Table S1). This article is the first time that the phenyl-glycosides and fat glycosides from the fruits of *Nicandra physaloides* have been studied and reported. Here, the isolation and structural elucidation of compounds **1**–**18**, as well as their anti-inflammatory activities, were elaborated and provided.

## 2. Results

### 2.1. Structure Elucidation

**Figure 2:**
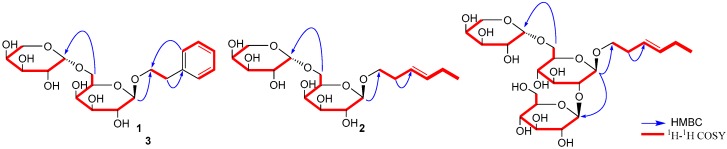
Key HMBC and ^1^H-^1^H COSY correlations of compound **1**–**3.**

Compound **1** possessed the molecular formula of C_19_H_28_O_10_ according to the HR-ESI-MS at *m*/*z* 417.1755 [M + H]^+^. The ^1^H-NMR spectrum (Table 1) of 1 showed the characteristic signals of mono-substituted benzene ring at δ_H_ 7.24 (4H, overlap, H-2, 3, 5, 6) and 7.16 (m, H-4). In addition, two protons signals of anomeric carbon of β-galactose and α-arabinose were observed at δ_H_ 4.65 (d, *J* = 7.9 Hz, H-1′) and 4.29 (d, *J* = 6.8 Hz, H-1′′), respectively. Combined with the DEPT, ^13^C-NMR spectrum of **1** showed 19 signals, including a group of benzene at δ_C_ 140.2, 130.0 (2 × C), 129.4 (2 × C) and 127.2, two methylene signals at δ_C_ 71.8 and 37.3, and two groups of galactopyranose and arabinopyranose glycosyls at δ_C_ 102.0, 72.3, 72.8, 68.8, 74.5, 69.8 and 105.2, 72.4, 74.2, 69.5, 66.7. The HMBC correlations (Figure 2) between H-1′ and C-8, H-1′′ and C-6′ suggested the attachment position of the galactosyl at C-8 and C-1′′ of arabinose at C-6′ of galactosyl [24]. Assignments of all groups of **1** were achieved by ^1^H-^1^H COSY, HSQC and HMBC (Figure 2). The absolute configuration of the glycosyls group of compound **1** was determined by GC analysis according to the same *t*_R_ at 12.9 and 32.5 min with standard l-arabinopyranose and d-galactopyranoside, respectively. From the above data, the structure of **1** was elucidated as 2-phenylethyl *O*-α-l-arabinopyranosyl-(1→6)-β-d-galactopyranoside, named Nicglycoside A.

Compound **2** was obtained as an amorphous powder with the molecular formula assigned as C_17_H_30_O_10_ by positive HR-ESI-MS from the [M + H]^+^ signal at *m*/*z* 395.1901, indicating 3 degrees of unsaturation The ^1^H-NMR spectrum (Table 1) of **2** showed two groups characteristic signals of galactose and arabinose glycosyls at δ_H_ 4.61 (1H, d, *J* = 8.0 Hz, H-1′) and 4.30 (1H, d, *J* = 6.7, H-1′′), which were in accordance with the glycosyl of compound **1**; a pair signals of double bonds δ_H_ 5.42 (2H, m), three methylene at δ_H_ 2.07 (2H, m), 2.37 (2H, m), 3.52(1H, o), 3.84 (1H, o) and one methyl at δ_H_ 0.96 (3H, t, 7.6). In the ^13^C-NMR, the glycosyl signals of galactopyranose and arabinopyranose existed at δ_C_ 102.0, 72.3, 72.8, 68.8, 74.4, 69.8 and 105.2, 72.4, 74.2, 69.5, 66.7. The HMBC correlations (Figure 2) between H-1′ and C-6, H-1′′ and C-6′ suggested the attachment position of the galactosyl was at C-6 and C-6′ of galactosyl was substituted connecting with the C-1′′ of arabinose. Its absolute configurations of the glycosyls group were determined by GC analysis as with the compound **1**. From the above data and combined with the literatures [24,25], the structure of **2** was elucidated as (*Z*)-hex-3-en-1-ol-α-l-arabinopyranosyl-(1→6)-β-d-galactopyranoside, named Nicglycoside B.

Compound **3** possessed the molecular formula of C_23_H_40_O_15_ according to the HR-ESI-MS at *m*/*z* 557.2419 [M + H]^+^. The ^1^H-NMR spectrum (Table 1) of **3** showed three characteristic signals of glycosyls at δ_H_ 4.59 (1H, d, *J* = 7.8 Hz, H-1′), and 4.30 (1H, d, *J* = 6.7, H-1′′), and 4.43 (1H, d, *J* = 7.6, H-1′′′), assigned to glucose, arabinose and glucose, respectively. Moreover, a group of parent nucleus signals existed in accordance with **2** at δ_H_ 5.42 (2H, m), 2.08 (2H, m), 2.36 (2H, m), 3.52 (1H, o), 3.84 (1H, o) and 0.97 (3H, t, 7.6). Combined with the DEPT, ^13^C-NMR spectrum of **3** showed 23 signals, including a fatly chain group, which is the same as compound **2**, at δ_C_ 14.7, 21.6, 134.5, 126.0, 28.8, 70.6, and two groups of glucose and a group of arabinose glycosyls at δ_C_ 105.0, 83.0, 78.2, 71.4, 77.7, 62.7, 103.0, 76.0, 77.7, 71.4, 76.7, 69.5 and 105.1, 72.4, 74.2, 69.5, 66.7. The HMBC correlations between H-1′ and C-6, H-1′′ and C-6′, and H-1′′′ and C-2′ suggested the attachment position of the arabinosyl at C-8 and C-1′′ of glucose at C-6′ of glucose. Assignments of all groups of **3** were achieved by ^1^H-^1^H COSY, HSQC and HMBC (Figure 2). The absolute configuration of the glycosyls group of compound **3** was determined by GC analysis according to the same *t*_R_ at 16.8 min and 12.9 min with standard d-glucose and l-arabinose, respectively. From the above data and combined with the literature [26,27], the structure of **3** was elucidated as (*Z*)-3-hexenyl *O*-β-d-glucopyranosyl-(1→2)-*O*-α-l-arabinopyranosyl-(1→6)-*O*-β-d-glucopyranoside, named Nicglycoside C.

### 2.2. Anti-Inflammatory Activity

All compounds **1**–**18** were evaluated for their anti-inflammatory activities (Table 2) inhibiting NO production of LPS-induced RAW 264.7 cells in vitro [27]. NO, as a key pro-inflammatory mediator, could suppress inducible enzyme expression via inhibition of the mitogen-activated protein kinase pathway and nuclear translocation of critical transcription factors [28,29]. The results suggested that the isolated compounds (**1**–**18**) possessed different degrees of activities in inhibiting NO production. For the compounds with the same mother nucleuses (**1**, **5**, **6**, **7**, **9**, **11**), compounds **7** and **11** showed weaker activities than others, which might be due to the moieties of rhamnose or trisaccharide. Compounds **8** and **10** showed weaker activities than other phenylethanoid glycosides, which might be ascribed to the hydroxy-substituted benzene rings.

## 3. Experimental Section

### 3.1. General Experimental Procedures

UV spectra were recorded on a Shimadzu UV-1601 instrument. HR-ESI-MS was performed on a Waters Xevo-TOF-MSTM.1D and 2D NMR spectra using a Bruker DPX 400 instrument with TMS as an internal standard. Preparative HPLC (Waters, Milford MA, USA, 515-2414) was performed on Sunfire (10 μm, 19 × 250 mm, Waters). ODS was obtained from YMC Company Ltd., Japan. Silica gel was used Qingdao Marine Chemical Ltd., Qingdao, China. All the solvents were of analytical grade and were purchased from Tianjinfuyu Company Ltd., Tianjin, China. ELISA reader was used from PerkinElmer, Waltham, MA, USA. The RAW 264.7 cells were from China Center for Type Culture Collection in Wuhan University, Hubei, China. DMEM was purchased from Corning, New York, NY, USA. The integrant biological agents were prepared, such as, MTT (Biotopped, Beijing, China), LPS (Sigma-Aldrich, St. Louis, MO, USA), DMSO (Sigma-Aldrich), Penicillin Streptomycin Solution (Corning, New York, NY, USA), Fetal bovine serum (Sijiqing, Hangzhou, China), NMMA (Sigma-Aldrich), Sulfanilic acid anhydrous (Tianli, Tianjin, China), *N*-(1-naphthyl) enylenediamine dihydrochloride (Damao, Tianjin, China), PBS (Biotopped).

### 3.2. Plant Material

The fruits of *Nicandra physaloides* (L.) Gaertn were harvested from Harbin, Heilongjiang Province of China, in September 2014, which was identified by Prof. Ruifeng Fan of Heilongjiang University of Chinese Medicine. The voucher specimen (20140911) had been deposited at Heilongjiang University of Chinese Medicine.

### 3.3. Extration and Isolation

The dry fruits (15 kg) of *Nicandra physaloides* were extracted with 70% ethanol 3 times and the condensed crude (1167.2 g) was fractioned by AB-8 macroporous resin column chromatography and eluted with 10% EtOH (439.5 g), 30% EtOH (209.4 g) and 95% EtOH (176.1 g). The 10% EtOH elution (100.0 g) was concentrated and separated. Thirteen obtained fractions (Fr. I-XIII) were combined based on the TLCs. Fr. V was separated by ODS chromatography, eluted with H_2_O/MeOH (0:1 to 0:1), to afford Fr. V-1 to V-17. Fr. V-9 was subjected to ODS chromatography (H_2_O/MeOH, 1:0 to 0:1) to afford **3** (12 mg), **10** (8 mg) **12** (9 mg) and **15** (10 mg). Fr. V-16 was separated by ODS chromatography (H_2_O/MeOH, 1:0 to 0:1) to afford Fr. V-16-1 to V-16-10. Fr. V-16-2 was purified by to afford **2** (10 mg), **4** (8 mg), **5** (9 mg) and **17** (9 mg). Fr. VI was separated by ODS chromatography, eluted with H_2_O/MeOH (0:1 to 0:1), to afford Fr. VI-1 to VI-10. Fr. VI-5 were purified by preparative HPLC to afford **9** (11 mg), **13** (8 mg), **16** (12 mg) and **18** (10 mg). Fr. X was purified by ODS chromatography (H_2_O/MeOH, 1:0 to 0:1), to afford Fr. X-1 to X-15. Fr. X-11 and X-13 were repeatedly subjected to ODS chromatography (H_2_O/MeOH, 1:0 to 0:1) and then purified by preparative HPLC to afford **1** (12 mg), **6** (9 mg), **7** (10 mg), **8** (11 mg), **11** (9 mg) and **14** (9 mg), respectively.

Nicglycoside A (**1**). White amorphous powder. ${\left[\alpha \right]}_{D}^{25}$ −8.9 (*c* = 1.30, MeOH); IR (KBr) 3435, 2921, 1750, 1260 cm^−1^; UV (MeOH) λ_max_ 212, 258, 271 nm; ^1^H- and ^13^C-NMR (MeOH, 400, 100 MHz) data, see Table 1; HR-ESI-MS *m*/*z* 417.1755 [M + H]^+^ (calcd. for C_19_H_29_O_10_, 417.1761) (Figures S1 and S2).

Nicglycoside B (**2**). White amorphous powder. ${\left[\alpha \right]}_{D}^{25}$ −46.4 (*c* = 1.57, MeOH); IR (KBr) 1732, 1464, 1263, 710 cm^−1^; ^1^H- and ^13^C-NMR (MeOH, 400, 100 MHz) data, see Table 1; HR-ESI-MS *m*/*z* 395.1901 [M + H]^+^ (calcd. for C_17_H_31_O_10_, 395.1917) (Figures S3 and S4).

Nicglycoside C (**3**). White amorphous powder ${\left[\alpha \right]}_{D}^{25}$ −51.8 (*c* = 1.60, MeOH); IR(KBr) 1730, 1458, 1260, 704 cm^−1^; ^1^H- and ^13^C-NMR (MeOH, 400, 100MHz) data, see Table 1; HR-ESI-MS *m*/*z* 557.2419 [M + H]^+^ (calcd. for C_23_H_41_O_15_, 557.2445) (Figures S5 and S6).

### 3.4. Acid Hydrolysis and GC Analysis

The isolated glycosides (**1**–**18**) (2.0 mg) were refluxed with 2 mL H_2_O and 2 N aqueous 1 mL HCl for water bath (3 h). Then, the reaction mixtures were extracted with ethyl acetate for 3 times (5 mL). The aqueous layer was neutralized and evaporated with MeOH and then dissolved in anhydrous pyridine (5 mL) and treated with l-cysteine methyl ester hydrochloride (1.5 mg). After being stirred for 1 h at 60 °C, the mixture was added into 150 μL of HMDS–TMCS (3:1) and then stirred for another 30 min at 60 °C. The supernatant was concentrated under N_2_ stream after being centrifuged off. The residue was partitioned between *n*-hexane and H_2_O (0.1 mL each), and the hexane layer (1 μL) was analyzed by GC [30,31], respectively. The configurations of d-glucose for compounds **3**–**18** were determined by the same *t*_R_ of standard d-glucose (*t*_R_ = 16.8 min), l-arabinose for compounds **1**–**5**, **16**, **18** (*t*_R_ = 12.9 min), d-galactose for compound **1**–**2** (*t*_R_ = 32.5 min), and l-rhamnose for compound **7**, **10** (*t*_R_ = 14.8 min).

### 3.5. Anti-Inflammatory Assays

RAW 264.7 cells were cultivated at densities of 5 × 10^5^ cell/wells in 96-well for 24 h, then discarded the supernatants and stimulated by LPS (100 μL, 1 μg/mL) to generate NO for cultivating 24 h. Following incubation of the demonstrated time, the amount of sable nitrite, the end product of NO generation by activated cells, were determined by a modification of the Griess reaction [32,33]. The cells were treated with 100 μL of the compounds by the final concentration of 5, 25, 50, 100 and 200 μg/mL. Briefly, 100 μL of culture supernatants from control or stimulated macrophages were transferred to 96-well plates. Supernatants were mixed with 50 μL of 1% sulfanilic acid anhydrous in 85% phosphoric acid, incubated for 10 min at room temperature, shielded from light, followed by 50 μL of 1 mg/mL *N*-(1-naphthyl) ethylenediamine dihydrochloride for 10 min incubation in light proof. The absorbance was measured at 540 nm using an ELISA reader, and nitrite concentration was determined by comparison with a sodium nitrite standard curve. NMMA was used as a positive control. No isolates did showed an effect on the assay systems with the final concentration 0.2 (*v*/*v*) in DMSO and the MTT assay revealed no significant cytotoxic effects (over 90% cells survival) on cells treated with above compounds at concentrations up to 200 μg/mL.

## 4. Conclusions

As described in the introduction, *Nicandra physaloides* possessed many kinds of bioactivities such as heat-clearing, detoxifying, antipyresis, and anti-inflammation, and was applied to the treatments of rheumatoid arthritis, and so on. This study obtained the 18 glycosides compounds from the *Nicandra physaloides* fruits, including phenyl-glycosides and fat glycosides. Meanwhile, many researchers have reported that the aromatic glycosides and phenyl-glycosides show significant anti-inflammatory activities [34,35,36,37]. Thus, the anti-inflammatory activities of the compounds were evaluated, some of which showed significant activities. These results indicated that these glycosides compounds could be the pharmacodynamic material basis for anti-inflammation from the *Nicandra physaloides* fruits, and played important roles in the treatments of inflammatory diseases such as rheumatoid arthritis, nasosinusitis, influenza, urinary tract infection, sore and furuncle. The systematic studies on the composition in this manuscript will be the foundations and references of further research. We have made contributions to discovering active ingredients and leading compounds and provided experimental and scientific basis of drug design and drug discovery of the *Nicandra physaloides*.

## Figures and Tables

**Table 1:** ^1^H- and ^13^C-NMR Data of **1**–**3** (CD_3_OD).

No.	1		2		3	
	δ_C_	δ_H_ mult (*J*, Hz)	δ_C_	δ_H_ mult (*J*, Hz)	δ_C_	δ_H_ mult (*J*, Hz)
1	140.2		14.6	0.96 (3H, t, 7.6)	14.7	0.97 (3H, t, 7.6)
2	129.4	7.24 overlap	21.6	2.07 (2H, m)	21.6	2.08 (2H, m)
3	130.0	7.24 overlap	134.5	5.42 (m)	134.5	5.42 (m)
4	127.2	7.16 (m)	125.9	5.42 (m)	126.0	5.42 (m)
5	130.0	7.24 overlap	28.8	2.37 (2H, m)	28.8	2.38 (2H, m)
6	129.4	7.24 overlap	70.6	3.52 overlap	70.6	3.54 overlap
				3.84 overlap		3.85 overlap
7	37.3	2.92 (t, 7.4)				
8	71.8	3.74 overlap				
		4.07 overlap				
1′	102.0	4.65 (d, 7.9)	102.0	4.61 (d, 8.0)	105.0	4.59 (d, 7.8)
2′	72.3	3.34 overlap	72.3	3.51 (m)	83.0	3.20–3.80 (m)
3′	72.8	4.04 (m)	72.8	4.05 (m)	78.2	3.20–3.80 (m)
4′	68.8	3.55 (m)	68.8	3.55 (m)	71.4	3.20–3.80 (m)
5′	74.5	3.83 (m)	74.4	3.82 (m)	77.7	3.20–3.80 (m)
6′	69.8	4.09 overlap	69.8	4.06 (dd, 11.2, 2.0)	69.5	4.09 (dd, 11.2, 1.9)
		3.70 overlap		3.72 (dd, 11.2, 5.0)		3.70 (dd, 11.4, 5.1)
1″	105.2	4.29 (d, 6.7)	105.2	4.30 (d, 6.7)	105.1	4.30 (d, 6.7)
2″	72.4	3.57 (m)	72.4	3.59 (m)	72.4	3.20–3.80 (m)
3″	74.2	3.47 (m)	74.2	3.50 (m)	74.2	3.20–3.80 (m)
4″	69.5	3.78 (m)	69.5	3.80 (m)	69.5	3.20–3.80 (m)
5″	66.7	3.50 (dd,12.5,3.2)	66.7	3.52 overlap	66.7	3.51 overlap
		3.85 (dd,12.5,2.0)		3.87 overlap		3.87 overlap
1″′					103.0	4.43 (d, 7.6)
2″′					76.0	3.20–3.80 (m)
3″′					77.7	3.20–3.80 (m)
4″′					71.4	3.20–3.80 (m)
5″′					76.7	3.20–3.80 (m)
6″′					62.7	3.52 overlap
						3.87 overlap

**Table 2:** Inhibitory on NO production in LPS-induced RAW 264.7 cells of compounds **1**–**18**.

Compounds	IC_50_ (μM)	Compounds	IC_50_ (μM)
NMMA ^b^	19.6 ± 2.4		
Compound **1**	31.1 ± 3.5	Compound **10**	>50
Compound **2**	32.9 ± 5.6	Compound **11**	41.7 ± 7.6
Compound **3**	41.2 ± 4.1	Compound **12**	29.8 ± 5.7
Compound **4**	30.2 ± 4.7	Compound **13**	36.6 ± 3.9
Compound **5**	26.9 ± 5.1	Compound **14**	>50
Compound **6**	37.5 ± 4.7	Compound **15**	25.1 ± 4.4
Compound **7**	41.2 ± 6.6	Compound **16**	38.9 ± 5.9
Compound **8**	>50	Compound **17**	33.4 ± 2.7
Compound **9**	34.8 ± 6.3	Compound **18**	31.4 ± 4.2

IC_50_ was defined as the concentration that resulted in a 50% inhibition on NO production. The IC_50_ greater than 50 μM was deemed inactive. ^b^ Positive control.

## Supplementary Materials

The following are available online: Figures S1–S6 and Table S1: The ^13^C-NMR data of Compounds **4**–**18**.

## Abbreviations

The following abbreviations are used in this manuscript:

HR-ESI-MS	High-resolution electrospray ionization mass spectrometry
NMR	Nuclear magnetic resonance
DEPT	Distortionless Enhancement by Polarization Transfer
HMBC	Heteronuclear multiple bond correlation
1H-1H COSY	Correlation spectroscopy
HSQC	Heteronuclear multiple quantum coherence
GC	Gas chromatography
*t*_R_	Retention time
NMMA	NG-monomethyl Arginine
UV	Ultraviolet
TMS	Tetramethylsilane
HPLC	High performance liquid chromatography
ODS	Octadecylsilyl
ELISA	Enzyme-linked immunosorbent assay
DMEM	Dulbecco’s modified eagle medium
MTT	3-(4,5-Dimethylthiazol-2-yl)-2,5-diphenyltetrazolium bromide
LPS	Lipopolysaccharides
DMSO	Dimethyl sulfoxide
PBS	Phosphate buffer saline
EtOH	Ethyl alcohol
TLC	Thin Layer Chromatography
MeOH	Methanol
HMDS-TMCS	Hexamethyldisi lazane-trimethylchlorosilane
