# Supplementary material for: New Glycosides from the Fruits of Nicandra physaloides
Source: Molecules. 2017 May 17;22(5):828. doi: 10.3390/molecules22050828 (PMC6154662; doi:10.3390/molecules22050828)
Supplement: Supplementary file 1 [file molecules-22-00828-s001.pdf]

Supporting Information

New glycosides from the fruits of *Nicandra Physaloides*

Yan Liu, HaiBing Jiang, ZhenPeng Xu, YanGang Cheng, ShaoWa Lv, BingYou Yang, HongWei Guo*, HaiXue Kuang*

Key Laboratory of Chinese Materia Medica, Heilongjiang University of Chinese Medicine, Ministry of Education, Harbin 150040, People's Republic of China

Corresponding Author

* hongweiguo17@163.com (H.-W.G.); hxkuang@hotmail.com (H.-X.K.); Tel.: +86-451-82193007 (H.-W.G.); +86-451-87267188 (H.-X.K.)

Caption:

Figure.S1. ^{1}H-NMR Spectrum of Compound **1**

Figure.S2. ^{13}C-NMR Spectrum of Compound **1**

Figure.S3. ^{1}H-NMR Spectrum of Compound **2**

Figure.S4. ^{13}C-NMR Spectrum of Compound **2**

Figure.S5. ^{1}H-NMR Spectrum of Compound **3**

Figure.S6. ^{13}C-NMR Spectrum of Compound **3**

Table.S1. The ^{13}C-NMR data of Compounds **4-18**

Figure.S1. ^{1}H-NMR Spectrum of Compound **1**

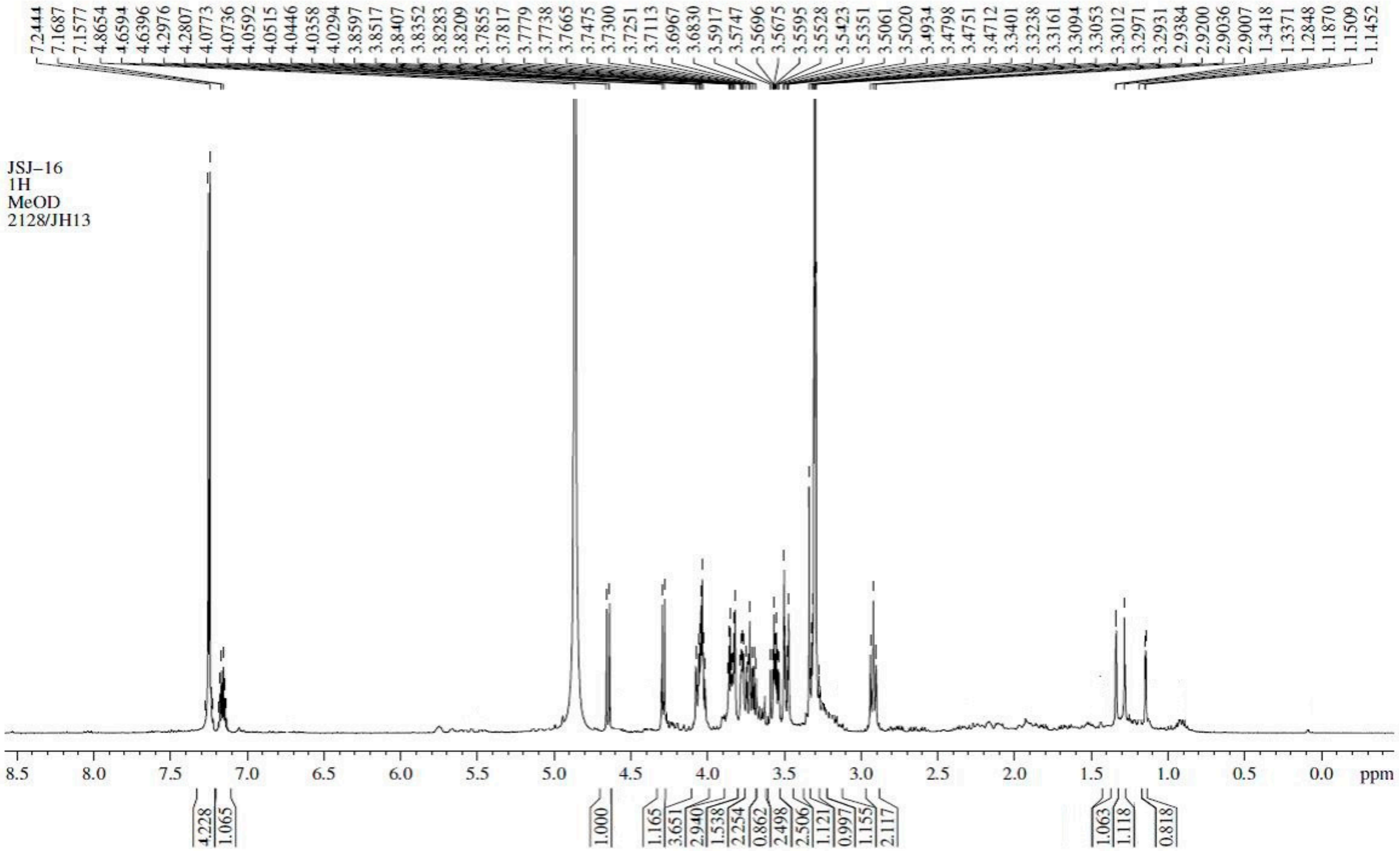

Figure.S2. ^{13}C-NMR Spectrum of Compound **1**

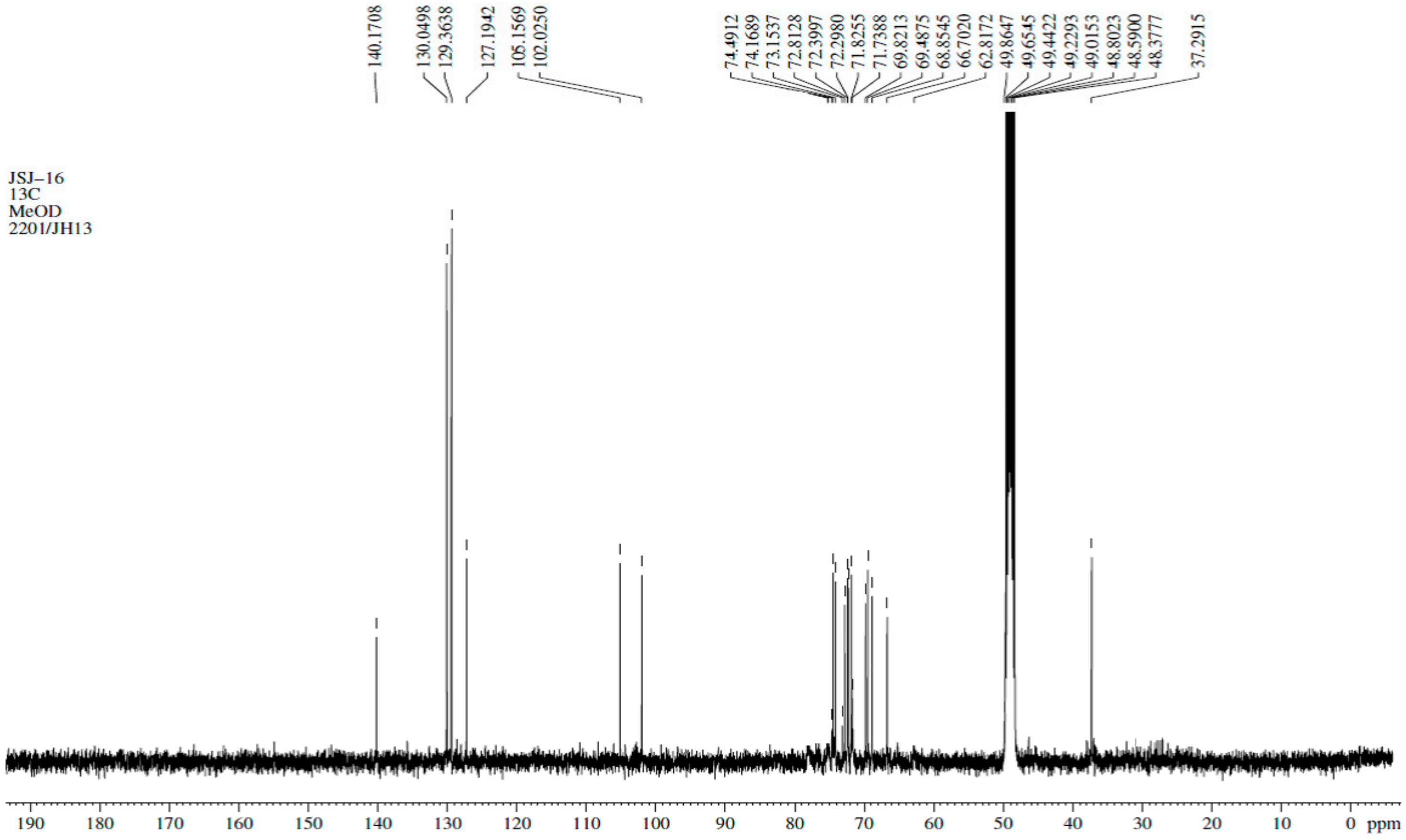

Figure.S3. ^{1}H-NMR Spectrum of Compound **2**

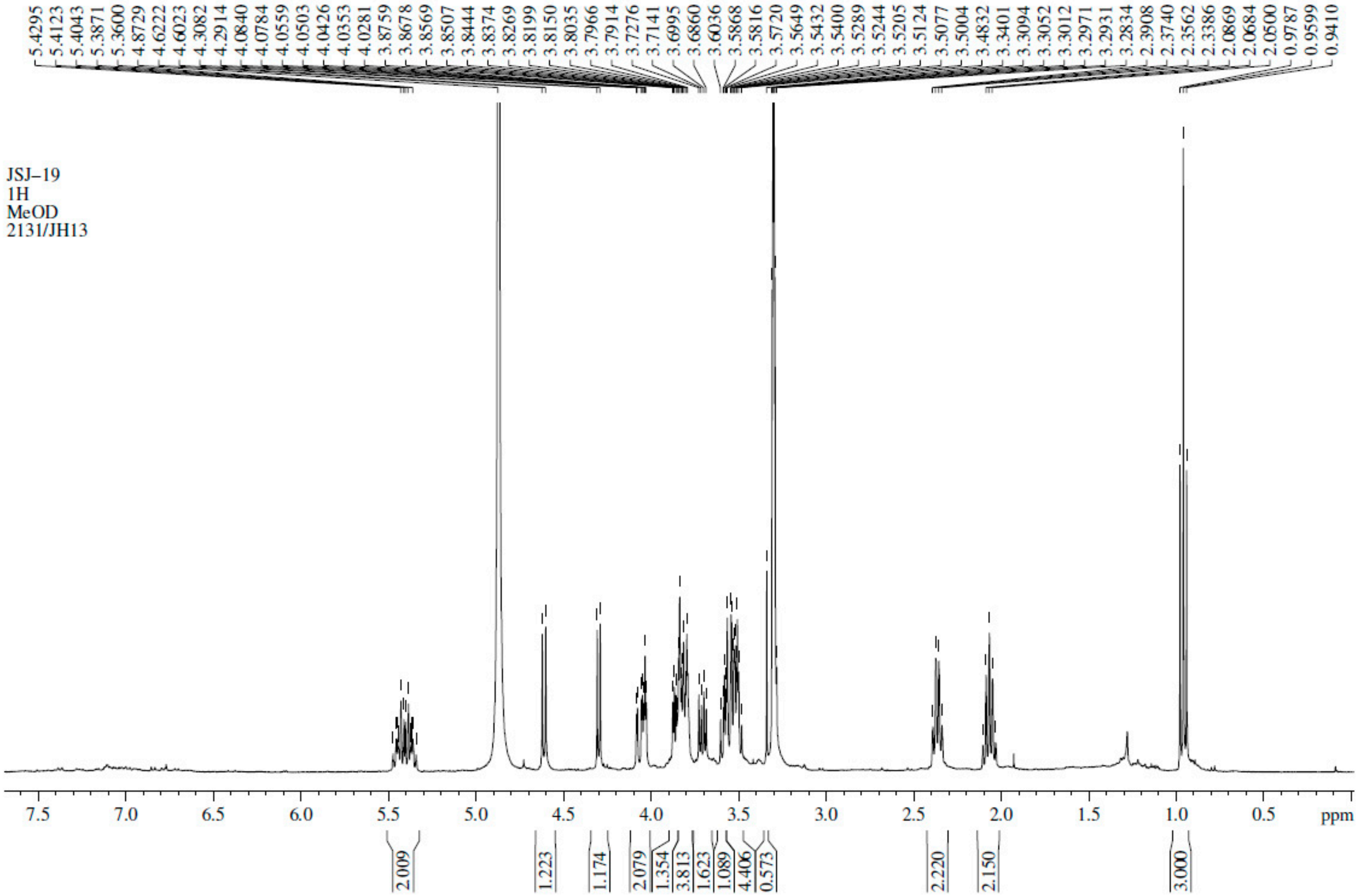

Figure.S4. ^{13}C-NMR Spectrum of Compound **2**

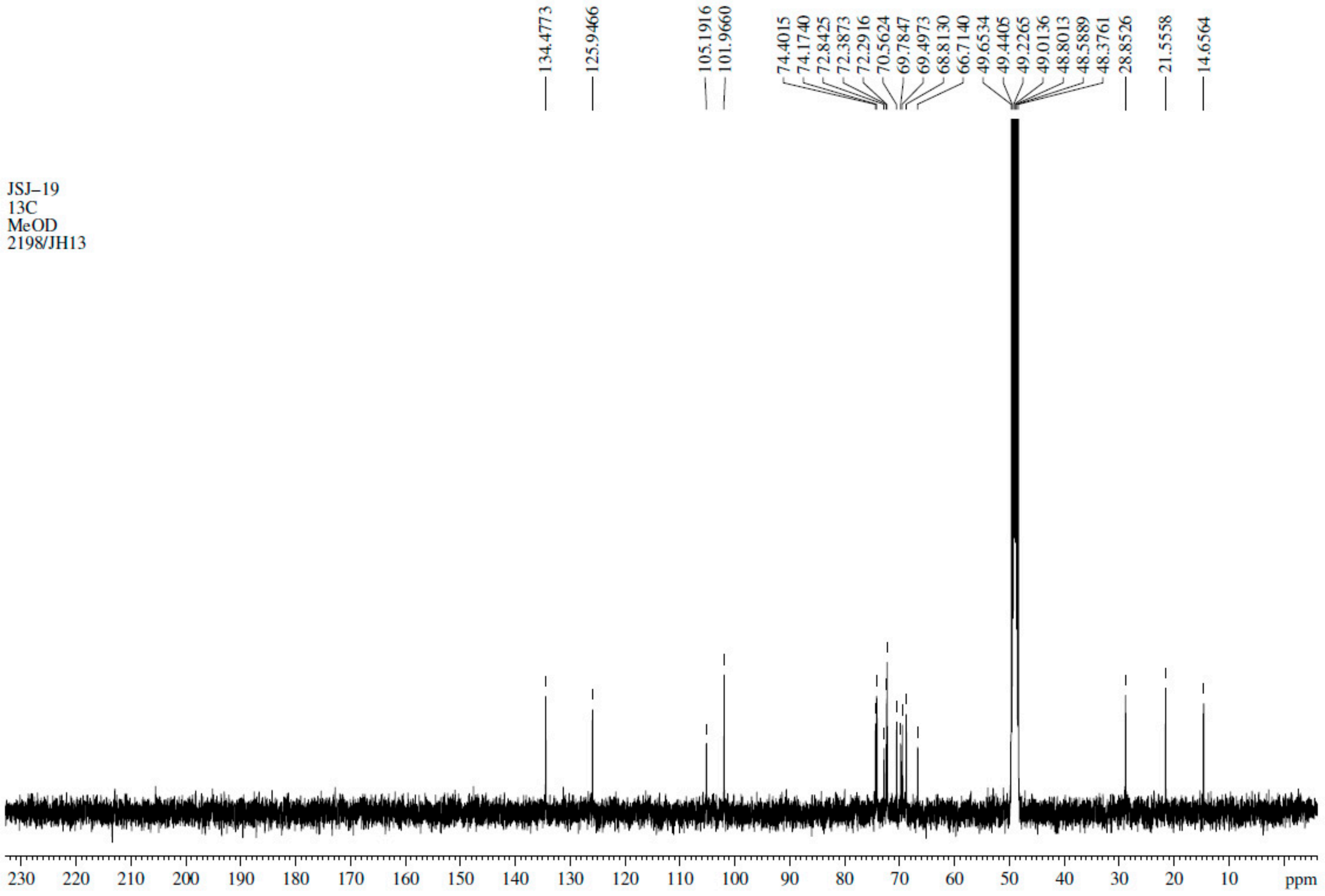

Figure.S5. ^{1}H-NMR Spectrum of Compound **3**

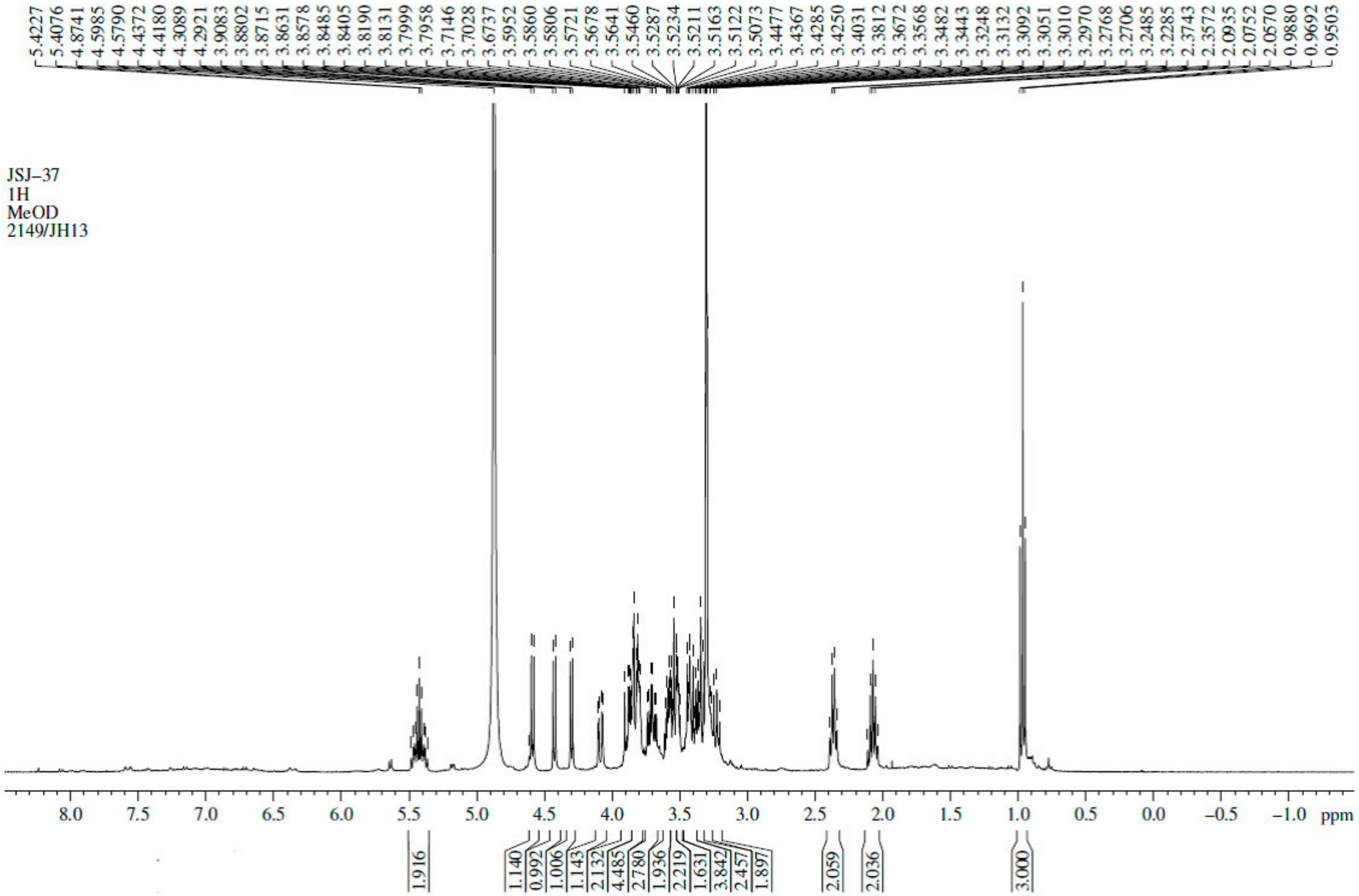

Figure.S6. ^{13}C-NMR Spectrum of Compound **3**

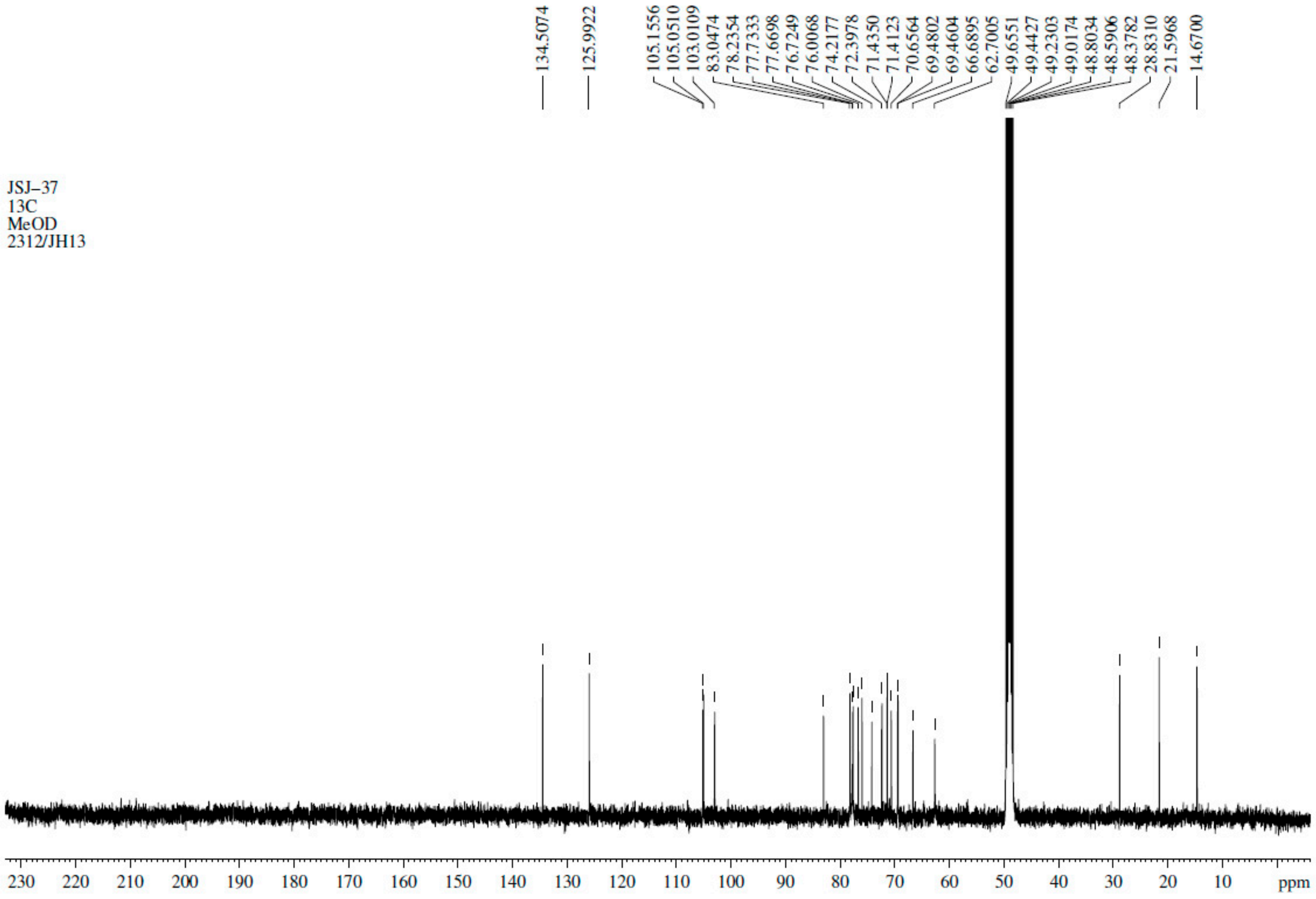
JSJ–37
13C
MeOD
2312/JH13
134.5074
125.9922
105.1556
105.0510
103.0109
83.0474
78.2354
77.7333
77.6698
76.7249
76.0068
74.2177
72.3978
71.4350
71.4123
70.6564
69.4802
69.4604
66.6895
62.7005
49.6551
49.4427
49.2303
49.0174
48.8034
48.5906
48.3782
28.8310
21.5968
14.6700
230 220 210 200 190 180 170 160 150 140 130 120 110 100 90 80 70 60 50 40 30 20 10 ppm

Table.S1. The ^{13}C-NMR data of Compounds **4-18** (100 MHz in ^{13}C NMR, MeOD)

**No.**	**4**	**5**	**6**	**7**	**8**	**9**	**10**	**11**	**12**	**13**	**14**	**15**	**16**	**17**	**18**
**1**	139.1	140.2	140.0	140.2	130.4	140.0	131.4	140.2	163.4	132.6	130.7	70.6	69.5	68.9	14.7
**2**	129.2	129.4	129.4	130.0	116.1	129.4	117.1	130.2	117.2	117.3	131.0	32.9	39.7	27.3	21.6
**3**	129.3	130.1	130.1	129.4	131.4	130.1	146.1	129.4	131.6	131.7	129.7	20.2	26.0	133.0	134.5
**4**	128.7	127.3	127.2	127.2	156.9	127.2	144.7	127.2	132.5	163.1	134.8	14.3	23.0	124.4	125.9
**5**	129.3	130.1	130.1	129.4	131.4	130.1	116.4	129.4	131.6	131.7	131.0		23.1	20.0	28.8
**6**	129.2	129.4	129.4	130.0	116.1	129.4	121.3	130.2	117.2	117.3	129.7				70.6
**7**	71.9	37.2	37.2	37.2	40.0	37.2	36.7	37.2	193.1	199.5	166.6				
**8**		71.9	71.9	71.9	73.2	71.9	71.7	71.9		26.5					
**1'**	103.4	104.4	104.4	101.6	104.9	104.4	104.5	103.0	99.5	101.6	96.3	104.4	104.4	100.4	104.4
**2'**	75.1	75.0	75.1	72.3	75.2	75.0	75.1	82.9	73.0	74.8	74.0	75.2	75.1	74.0	75.0
**3'**	77.9	77.9	78.0	72.4	77.9	77.9	78.1	77.7	72.0	77.9	77.9	77.9	78.0	71.4	78.0
**4'**	71.5	71.5	71.7	73.7	71.6	71.4	72.2	71.4	68.6	71.3	70.9	71.9	71.6	70.9	71.5
**5'**	77.0	76.9	78.1	69.8	77.9	77.0	76.8	76.8	75.8	78.3	78.0	78.2	76.8	67.5	76.8
**6'**	69.6	69.6	62.8	18.0	62.6	69.8	68.1	69.5	62.8	62.5	69.5	62.8	69.4	61.6	69.5
**1''**	105.2	105.2				104.8	102.2	105.2			101.9		105.2		105.2
**2''**	72.4	72.4				75.1	72.4	76.7			75.1		72.4		72.4
**3''**	74.2	74.2				78.0	72.3	78.2			78.0		74.2		74.2
**4''**	69.6	69.6				71.6	74.0	71.5			71.5		69.4		69.5
**5''**	66.8	66.8				78.0	69.8	78.2			77.9		66.7		66.8
**6''**						62.8	18.1	62.8			62.6				
**1'''**								103.0							
**2'''**								72.4							
**3'''**								74.2							
**4'''**								69.5							
**5'''**								66.7							
